# Evaluation of a project to engage patients in the development of a patient‐reported measure for HIV care (the I‐Score Study)

**DOI:** 10.1111/hex.12845

**Published:** 2018-10-29

**Authors:** David Lessard, Kim Engler, Isabelle Toupin, I‐Score Consulting Team, Jean‐Pierre Routy, Bertrand Lebouché

**Affiliations:** ^1^ Department of Family Medicine McGill University Montreal Quebec Canada; ^2^ Research Institute of the McGill University Health Centre (MUHC) Montreal Quebec Canada; ^3^ Royal Victoria Hospital, Chronic and Viral Illness Service (MUHC) Montreal Quebec Canada; ^4^ SPOR Mentorship Chair in Innovative Clinical Trials of the CIHR Montreal Quebec Canada

## Abstract

**Background:**

Patient engagement (PE), patients’ meaningful involvement in research through partnerships and sensitivity to their expertise, is receiving attention. However, PE initiatives are poorly reported and little is known about patients’ perspective on PE.

**Objective:**

To document and evaluate the first phase (22 months) of a PE Project for the I‐Score Study which is developing a patient‐reported measure of HIV treatment adherence barriers, we describe the nature of PE conducted, determine the level of PE achieved and present its impacts from the engaged patients’ perspective.

**Setting and participants:**

A Montreal‐based committee of ten people with HIV was recruited from community and clinical settings and participated in: I‐Score study decision making, knowledge dissemination, research on the experience of people with HIV and the PE project's evaluation.

**Methods:**

The evaluation followed a convergent parallel mixed‐methods design. Data collection included participant observation, a satisfaction survey and meeting minutes/transcriptions. Analysis entailed reporting PE activities, generating descriptive statistics and thematically analysing qualitative material.

**Results:**

PE consisted of twelve meetings, including two focus groups (needs assessment), in addition to four knowledge dissemination activities. PE levels showed an increase: the first four regular meetings entailed information/consultation, while subsequent meetings reached implication/collaboration. Regarding impacts, patients indicated high and stable satisfaction rates (*M *=* *4.4/5; SD = 0.76). Furthermore, thematic analysis identified “positive interactions,” “co‐learning,” “self‐determination,” and “the collective management of confidentiality” as important PE impacts for engaged patients.

**Conclusion:**

This PE Project evaluation highlighted growing engagement levels, high satisfaction rates and the importance of a patient‐centric approach to PE.

## 1 INTRODUCTION

Patient engagement (PE) refers to patients’ meaningful involvement in potentially all steps of research to account for their expertise and perspective relative to their health condition, treatments and care.^1^ PE represents a shift^2^ emphasizing: the importance of values and deliberation in health‐related decision making;^3^, ^4^ patients’ autonomy;^5^ sensitive listening and accountability to their circumstances; and partnerships.^6^ Given that PE treats patients as actors in research and of their own care, it involves joint action and co‐construction of knowledge to empower patients, democratize knowledge and reduce paternalism in health care.^7^, ^8^, ^9^, ^10^

Several frameworks^7^, ^9^, ^11^, ^12^, ^13^, ^14^, ^15^, ^16^, ^17^ are available to guide its practice. PE has been conceived as a spectrum ranging from: (a) information (informing patients); (b) consultation (obtaining patients’ perspective); (c) implication (accounting for patients’ concerns in decision making); and (d) collaboration (partnering with patients in decision making); to (e) empowerment (placing decisions in patients’ hands).^16^, ^18^, ^19^ PE has broad applicability in research; it is used with different: health conditions (cancer, chronic pain, diabetes, etc.), populations (eg, older people), interventions (eg, physiotherapy, surgery), settings (eg, homelessness, community) and methodologies (eg, systematic review, health technology development).^20^, ^21^ It is also gaining greater attention, in part, as it is reported to improve the quality of research and care.^7^, ^8^, ^10^, ^11^, ^22^, ^23^

There have been calls since the beginning of the 1980s to involve people living with HIV (PLHIV) in all aspects of the response to the epidemic,^24^, ^25^ through PE, among other practices. Facilitators of PE with PLHIV include: direct communication between engaged PLHIV, care providers, or investigators,^26^ active listening to PLHIV's concerns, and emphasis on patient‐tailored health‐related information PLHIV can use in their daily lives.^27^ Challenges noted by UNAIDS^24^ and investigators^28^ include social and gender inequalities, concerns about disclosure and felt stigma.^24^, ^28^ Greater consideration of how engaged PLHIV perceive PE or tackle these challenges is needed.^29^

Methodologically, several limitations of PE have been raised. For instance, it is often unclear what process or model of PE was applied.^10^, ^30^, ^31^ PE evaluation designs and methods are generally inadequately described^31^, ^32^, ^33^ and many aspects of PE initiatives are underreported, including recruitment, participation rate, patient satisfaction, frequency or details of PE activities, and impacts.^32^, ^34^, ^35^ Overall, few studies have incorporated the patient perspective in PE evaluation.^35^, ^36^, ^37^, ^38^ Doing so is important as investigators and patients can disagree on patients’ functions^35^ which may negatively impact patients’ retention^39^ and satisfaction with PE.^37^, ^40^ Furthermore, in Canada, PE uptake remains slow and identifying ways to increase benefits for patients is needed to optimize their enrolment and retention in PE.^33^ Involving patients in PE evaluation and gaining their perspective on PE could help.^35^

To address these concerns, this paper's objectives are to document, in detail, and evaluate a PE Project's first phase (November 2015‐September 2017), reporting: 1) the nature of PE conducted, 2) levels of PE achieved and 3) its impacts from the perspective of engaged PLHIV.

## 2 METHODS

### 2.1 Patient engagement project context

This article focuses on a PE Project (hereafter, the Project) designed for the I‐Score Study, a study launched in January 2016 to create, validate and integrate into HIV clinical practice an electronic patient‐reported outcome measure (PROM) of antiretroviral treatment (ART) adherence barriers. Its rationale and exploratory multi‐method (qualitative and quantitative) four‐step design are explained elsewhere.^41^ So far, I‐Score investigators have completed its first step, that is, the generation of the PROM's conceptual framework and its items, informed by qualitative interviews with PLHIV, a review of HIV‐specific PROMs^42^ and a synthesis of qualitative research with PLHIV on ART adherence barriers.^43^

### 2.2 Patient engagement project rationale

The Project was initiated when I‐Score Study investigators realized that the PROM's success depended on evidence of its value to PLHIV^44^ and other stakeholders.

The I‐Score investigators chose a mode of PE consisting of an advisory committee of PLHIV, which promised direct, equitable and continuous engagement of patients in decision making throughout the Study. Such continuous partnerships are reported to optimize research outcomes of PE.^10^, ^31^ In addition, investigators understood that combining PE with participation in research could add value to PE.^45^, ^46^, ^47^ However, it is important to clearly distinguish PE activities from engaged patients’ participation in research.^47^ Hence, investigators attributed three main functions to the committee. Members were:

1. *Stakeholders in decision making* about the I‐Score Study. This implied attending meetings consisting of deliberative discussions^48^ to make recommendations to investigators on issues raised while conducting the Study and being involved in the evaluation of the Project.
2. *Actors in knowledge dissemination*. This consisted of collaborating with investigators in the organization/presentation of knowledge dissemination activities (KDAs) to disseminate I‐Score Study research results in the HIV, health‐care provider or academic communities.
3. *Participants in qualitative and quantitative research*. This included participation in data collection activities to document their perspective on the Project and issues relevant to the I‐Score Study.

This article reports on the evaluation of the PE Project during its first phase (November 2015‐September 2017).

Ethics approval is generally not required for PE.^45^, ^49^ However, because engaged patients were involved as participants in research, the Project was submitted to the Research Ethics Board of the Research Institute of the MUHC which approved it on 8 September 2015. Thus, engaged patients had to give their informed consent. The investigators also decided to financially compensate them to recognize their contributions (50 CAD per participant, per meeting or KDA) and because monetary incentives have positive impacts on PLHIV's well‐being.^50^

### 2.3 Patient engagement project implementation: forming the I‐Score Consulting Team

The advisory committee, subsequently renamed by members as the I‐Score Consulting Team (henceforth, the Team), was formed in October 2015. Drawing on qualitative research methods, investigators used a maximum variation sampling technique, which aims to capture a wide range of perspectives.^51^ Sampling sought to include PLHIV with: different durations of ART use (three months minimum), varying levels of experience with HIV research or community organizing and abilities to share experiences in a group.^52^ We sought representation of different age groups and of the main groups affected by HIV in Quebec, that is, gay, bisexual, and other men who have sex with men (62% of PLHIV in Quebec), people from HIV endemic countries (15%), heterosexual women (10%) and people who inject drugs (4%).^53^ Investigators arbitrarily decided that the Team would include a maximum of ten members, all adults, based in Montreal, Canada. As in similar initiatives,^54^, ^55^ the chosen Team size reflected concerns for the viability of the Project: it seemed favourable to the development of meaningful relationships and to ensuring sufficient attendance.

To recruit Team members and obtain their informed consent, four Montreal‐based community organizations or initiatives with which I‐Score investigators had previously collaborated (AIDS Community Care Montreal, Portail VIH/Sida du Québec, Maison Plein Coeur, Projet PluriELLES) and health professionals at the McGill University Health Centre (MUHC) displayed flyers or suggested potential members. Interested individuals (n = 19) contacted DL and IT by phone. The Project's main objectives and general expectations (eg, to attend meetings, participate in KDAs) were explained. One person withdrew at this stage without explanation.

DL met in person with the remaining eighteen candidates to discuss in detail the conduct of meetings, the committee's functions and the anticipated composition of the Team, entering information into a logbook on: when they were diagnosed; when they initiated ART; the extent of their participation in community organizing or research; their sex, age, sexual orientation and country of origin; and their experience using injected drugs. He also explained and handed to candidates an informed consent document, summarizing the objectives of the Project, the committee's functions, anticipated results, potential risks and benefits, measures to ensure Team members’ confidentiality and the main investigators’ contact information. Finally, investigators selected Team members based on information provided during this meeting and asked the candidates to return the signed informed consent document at the first Team meeting.

Table 1 presents Team members’ characteristics.

**Table 1:** Characteristics of I‐Score Consulting Team members

Member^a^	Age group (in years)	Group	Number of years on ART	Reported experience in research/community organizing
01	30‐39	White MSM	3 to 6 years	Involvement in several HIV community organizations; experience in community‐based research
02	30‐39	White MSM	Over 10 years	Involvement in several community organizations
03	30‐39	European White MSM	Less than 1 year	Professional background in academic research; participant in clinical research
04	40‐49	African WSM	Over 10 years	Experience in community‐based research; participant in clinical research
06	50‐59	African WSM	Over 10 years	Involvement in an HIV community organization
07	50‐59	White WSM	1 to 3 years	None
08	50‐59	White WSM ex‐PWID	Over 10 years	Participant in clinical research
12	50‐59	White MSM	Over 10 years	Involvement in an HIV community organization
15	60‐69	African MSW	Over 10 years	Involvement in several HIV community organizations; participant in clinical research
16	20‐29	African woman self‐identified as queer	Over 10 years	Involvement in an HIV community organization

MSM, man who has sex with men; MSW, man who has sex with women; PWID, person who inject drugs; WSM, woman who has sex with men.

a Member number refers to the order in which individuals were recruited for consideration for the Team.

### 2.4 Patient engagement project evaluation

#### 2.4.1 Design

The evaluation of the PE Project followed a convergent parallel mixed‐methods design,^56^ combining qualitative and quantitative^57^ data collection and analysis methods, in order to confirm/complement and triangulate information using different datasets,^57^ and involving Team members in the co‐construction of knowledge.^58^, ^59^ While PE evaluation designs often combine different methods, there is no consensus on how to involve patients in PE evaluation.^35^

#### 2.4.2 Data collection

Several data collection methods were used, and all Team members were aware of these.

Through participant observation,^60^ with DL's work as a facilitator, detailed notes were taken on attendance, meeting duration, changes in facilitators, discussion themes, Team members’ functions, and, if applicable, their associated research activity and impacts on Team members. To identify discussions, after each meeting, DL examined his meeting notes in relation to the topics or tasks proposed in the meeting agenda, adjusting or adding such “themes” as necessary. The meeting minutes, organized by theme, were then transferred to members for validation.

As a part of collecting data on the patient perspective on PE, an anonymized satisfaction survey was used, which was inspired from instruments used in previous engagement initiatives.^61^, ^62^ The survey had two sections. A quantitative section allowed members to rate their satisfaction with a 5‐point Likert scale on different aspects of the meeting (see Table 2). A qualitative section asked open‐ended questions on elements of the meeting that went well/wrong and impacts of the Project, including elements that were considered important, were learned or could change their behaviour. Members filled out the survey on odd‐numbered Team meetings. At the end of even‐numbered meetings, DL also asked the Team to respond to the open‐ended survey questions in a group discussion.

**Table 2:** Team members’ satisfaction with features of the meetings^a^; average (range; standard deviation)^b^

Aspect	Meeting #3	Meeting #5	Meeting #7	Meeting #9	Meeting #11
Interest	4.3 (3‐5; 0.87)	4.7 (3‐5; 0.71)	4.6 (3‐5; 0.74)	4.6 (3‐5; 0.79)	4.6 (3‐5; 0.79)
Relevance	4.2 (3‐5; 0.97)	4.7 (3‐5; 0.71)	4.6 (3‐5; 0.74)	4.6 (3‐5; 0.79)	4.6 (3‐5; 0.79)
Enjoyment	4.3 (3‐5; 0.87)	4.7 (3‐5; 0.71)	4.6 (3‐5; 0.74)	4.4 (3‐5; 0.79)	4.6 (3‐5; 0.79)
Meeting with people	4.2 (3‐5; 0.83)	4.4 (3‐5; 0.73)	4.5 (3‐5; 0.76)	4.1 (3‐5; 1.00)	4.3 (4‐5; 0.53)
Learning new information	3.2 (2‐5; 0.87)	4.2 (2‐5; 1.20)	4.6 (3‐5; 0.74)	4.4 (3‐5; 0.98)	4.1 (3‐5; 0.69)
Learning new skills	3.8 (3‐5; 0.97)	4.2 (3‐5; 0.83)	4.6 (4‐5; 0.52)	4.6 (3‐5; 0.77)	4.3 (3‐5; 0.76)
Venue/facility	3.8 (1‐5; 1.30)	4.2 (3‐5; 0.97)	4.3 (3‐5; 0.71)	4.1 (3‐5; 0.69)	4.6 (3‐5; 0.77)
Event timing	3.9 (3‐5; 0.71)	4.3 (3‐5; 0.73)	4.6 (4‐5; 0.52)	4.0 (3‐5; 0.69)	4.1 (3‐5; 0.76)
Facilitation	4.7 (4‐5; 0.50)	4.7 (3‐5; 0.73)	4.8 (4‐5; 0.46)	4.9 (4‐5; 0.38)	4.7 (4‐5; 0.49)
Catering/refreshments	4.1 (3‐5; 0.93)	4.0 (3‐5; 0.97)	4.8 (4‐5; 0.46)	4.7 (4‐5; 0.49)	4.4 (3‐5; 0.79)
Global average per meeting	4.0 (1‐5; 0.91)	4.4 (3‐5; 0.82)	4.6 (3‐5; 0.63)	4.4 (3‐5; 0.75)	4.4 (3‐5; 0.70)

a Scale of 1 (completely unsatisfied) to 5 (completely satisfied).

b Members did not fill out the satisfaction survey during Meetings 1 and 2 because these were structured focus groups.

Each meeting was audio‐recorded (qualitative data) to complete observational notes and survey data, and document members’ perceived impacts of the Project.

#### 2.4.3 Data analysis

To describe the nature of PE, we entered meeting characteristics from observational notes into a Microsoft Word table and examined changes over time.

To determine the level of PE achieved, we referred to the International Association of Public Participation's spectrum of engagement,^16^, ^18^, ^19^ classifying each Team discussion along the continuum. This was done by considering observational notes, transcripts, tasks given to or determined by the Team members’ themselves, member functions and their overall influence in decision making. Attributed level of PE (see Table 3) was validated with the Team and other investigators.

To define impacts of PE from the patient perspective, we generated descriptive statistics for each quantitative satisfaction survey item. We also analysed member‐identified impacts by conducting an inductive thematic analysis^63^ of the qualitative responses to the satisfaction survey and of meeting transcripts. After multiple readings, DL coded the relevant material and generated themes^64^ which were assessed and discussed with other investigators for coherence and accuracy. During the two last meetings, they discussed their perceived impacts of the PE Project (Meeting 11, Discussion 34) and were involved in the PE evaluation by discussing the satisfaction survey results and the preliminary qualitative analyses (Meetings 12, Discussion 37). This helped ensure no impact was missed and allowed members to explain the results in their own words.^65^ Such respondent validation can limit biases, increase the trustworthiness of analyses and ensure the integrity of interpretations.^66^, ^67^

**Table 3:** Detailed description of I‐Score Consulting Team meetings, discussions and knowledge dissemination activities over the Patient Engagement Project evaluation period

Meeting details	Discussion theme	Members’ function (and research impacts when members’ function was to participate in data collection)	Duration	Concerned research activity and project	Impact for the Team	Level of engagement
Meeting #1 19 November 2015/2 h/4 members (women only)/Community Centre	1. ART adherence barriers that PLHIV would like to report to their clinician, and how they would like to report them	Participants in data collection Needs assessment for the I‐Score PRO measure and better understanding of PLHIV's perception of ART adherence barriers^70^	120 min	Needs assessment I‐Score Study	Structured discussion on experiences of ART and HIV; needs assessment for the I‐Score PRO measure	Consultation (Structured focus group)
Meeting #2 26 November 2015/2 h 15 m/5 members (men only)/Community Centre	2. ART adherence barriers that PLHIV would like to report to their clinician, and how they would like to report them	Participants in data collection Needs assessment for the I‐Score PRO measure and better understanding of PLHIV's perception of ART adherence barriers^70^	135 min	Needs assessment I‐Score Study	Structured discussion on experiences of ART and HIV; needs assessment for the I‐Score PRO measure	Consultation (Structured focus group)
Meeting #3 14 December 2015/2 h 23 m/9 members /MUHC	3. Members’ self‐introduction	Stakeholders in decision making	28 min	Functioning Patient Engagement Project	Introduction of each Team member	Information
	4. Overview of Team member selection, main objectives, and functioning of meetings	Stakeholders in decision making	42 min	Functioning Patient Engagement Project	Common understanding of the Project	Information
	5. Overview of the I‐Score Study's design	Stakeholders in decision making	9 min	Functioning I‐Score Study	Common understanding of the I‐Score Study	Information
	6. Proposed qualitative interview schedule for patients	Stakeholders in decision making	42 min	Validation of data collection instrument I‐Score Study	Spontaneous discussion on experiences of ART and HIV	Consultation
	Other		22 min	—	—	—
Meeting # 4 4 February 2016/2 h 41 m /9 members/Community Centre	7. Presentation of focus groups transcriptions (Meetings 1&2)	Stakeholders in decision making	16 min	Validation of data collection instrument Patient Engagement Project	Spontaneous discussion on experience of focus groups	Consultation
	8. Explanation of the proposed process of meeting evaluation by Team members	Stakeholders in decision making	4 min	Validation of data collection instrument Patient Engagement Project	Decision to anonymize the evaluation survey and to hold semi‐structured Team discussions on even‐numbered meetings	Implication
	9. Discussion about appropriate group name for members	Stakeholders in decision making	11 min	Functioning Patient Engagement Project	—	Implication
	10. Qualitative interview schedule with patients (continuation of Discussion 4)	Stakeholders in decision making	33 min	Validation of data collection instrument I‐Score Study	Spontaneous discussion on experiences of ART and HIV	Consultation
	11. Literature review of HIV‐specific patient‐reported outcomes	Participants in data collection Better understanding of PLHIV's perception of clinical PROMs^42^	45 min	Validation of results and interpretations I‐Score Study	Spontaneous discussion on members’ experiences of HIV and health care	Consultation
	12. Discussion of clinicians’ perceptions of patients’ needs	Participants in data collection Contextualization of results for Subproject 1 and better understanding of PLHIV's experience of care^71^	21 min	Validation of results and interpretations Subproject 1^a^	Spontaneous discussion on experience of care	Consultation
	Other		31 min			
Meeting #5 23 March 2016/2 h 58 m/9 members/Community Centre	13. Discussion about appropriate label for members (continuation of Discussion 7)	Stakeholders in decision making	6 min	Functioning Patient Engagement Project	Consensus on committee's title (I‐score Consulting Team)	Implication
	14. First qualitative interviews with HIV patients (continuation of Discussion 8)	Stakeholders in decision making	6 min	Validation of data collection instrument I‐Score Study	Follow‐up	Information
	15. History of HIV‐specific patient‐reported outcomes (continuation of Discussion 9)	Participants in data collection Better understanding of PLHIV's perception of clinical PROMs^42^	49 min	Validation of results and interpretations I‐Score Study	Spontaneous discussion on discrimination of PLHIV	Consultation
	16. Discussion on barriers and facilitators of patient‐clinician communication	Stakeholders in decision making Better understanding of PLHIV's experience of clinical care	50 min	Identification of research question Subproject 2^b^	Spontaneous discussion on patient‐clinician communication	Consultation
	17. Patients’ perspective on adherence (results of analysis of Meetings 1 & 2)	Participants in data collection Needs assessment for the I‐Score PRO measure and better understanding of PLHIV's perception of ART adherence barriers^70^	23 min	Validation of results and interpretations Patient Engagement t	Information provided on the process focus group analysis and preliminary^b^ results	Information
	18. Discussion of logos and designs for the digital application of the I‐Score PRO	Stakeholders in decision making	13 min	Design of digital application supporting the PRO measure I‐Score Study	Consultation on preferences concerning the digital application for the I‐Score PRO	Implication
	Other		31 min			
Meeting #6 18 May 2016/2 h 50 m/6 members/Community Centre	19. Members’ confidentiality in publications about the Patient Engagement Project	Stakeholders in decision making	28 min	Knowledge dissemination Patient Engagement Project	Discussion of appropriate ways to acknowledge members’ contribution to the research process	Implication
	20. Relevance of recommendations for the management of ART adherence barriers	Stakeholders in decision making	12 min	Application for funding/infrastructure Subproject 3^c^	Information on Recommendations Project^a^	Consultation
	21. Discussion of clinicians’ perceptions of patients’ reality	Participants in data collection Validation of results for Subproject 1 and better understanding of PLHIV's experience of care^72^	1 h 42 min	Validation of results and interpretations Subproject 1^a^	Spontaneous discussion on personal and common HIV‐ and ART‐related experiences	Consultation
	Other		28 min			
Meeting #7 7 September 2016 3 h/8 members/Community Centre	22. Information on recent developments	Stakeholders in decision making	17 min	Identification of research question Subproject 3^c^	Follow‐up	Information
	23. Organization of KDAs 1, 2 and 3	Actors in KDAs	1 h	Knowledge dissemination Patient Engagement Project	Discussion of potential themes; selection of participant members	Consultation
	24. Discussion of existing and potential interventions for adherence barriers	Stakeholders in decision making Better understanding of PLHIV's experience of clinical care	1 h 4 min	Identification of research question Subproject 3^c^	Spontaneous discussion on potential and actual interventions to manage ART adherence barriers	Consultation
	Other		39 min			
Meeting #8 21 September 2016/2 h 39 m/8 members/Community Centre	25. Patients’ perceptions of ART adherence barriers (results of analysis of Meetings 1 & 2)	Participants in data collection Needs assessment for the I‐Score PRO measure and better understanding of PLHIV's perception of ART adherence barriers^70^	2 h 21 min	Validation of results and interpretations Patient Engagement Project	Spontaneous discussion on ART adherence barriers	Implication
	Other		18 min			
Meeting #9 2 November 2016/2 h 43 m/7 members/Community Centre	26. Recent publications and presentations	Stakeholders in decision making	1 min	Academic publication process I‐Score Study	Summary of recent publications and presentations at conferences	Information
	27. Preparation of a letter of support for Mentorship Chair (application for infrastructure)	Stakeholders in decision making	1 h 14 min	Application for funding/infrastructure Subproject 4^d^	Discussion on members’ responsibilities and motivations	Implication
	28. Patients’ perceptions of ART adherence barriers (Continuation of Discussion 25)	Participants in data collection Needs assessment for the I‐Score PRO measure and better understanding of PLHIV's perception of ART adherence barriers^70^	17 min	Validation of results and interpretations Patient Engagement Project	Follow‐up	Information
	29. Preparation of testimonials for KDAs	Actors in KDAs	40 min	Knowledge dissemination Patient zngagement Project	Discussion over the content of testimonials	Implication (co‐facilitated by a member)
	Other		31 min			
Meeting # 10 14 December 2016/2 h 36 m/9 members/Community Centre	30. Discussion of KDA 3; preparation of KDA 4	Actors in KDAs	47 min	Knowledge dissemination Patient Engagement Project	Follow‐up of KDA 3, discussion on KDA 4, and selection of participants	Consultation
	31. Participation in clinical trials and experiences of ART switches	Stakeholders in decision making Better understanding of PLHIV's experience of clinical care	1 h 25 min	Identification of research question Subproject 2^b^	Spontaneous discussion on members’ concerns about participation in research projects and clinical trials	Collaboration (co‐facilitated by a member /research partner)
	Other		24 min			
Meeting # 11 22 March 2017/2 h 42 m/7 members/Community Centre	32. Information on the results of the application for the Mentorship Chair and changes to plan in the infrastructure of the Project	Stakeholders in decision making	4 min	Functioning Subproject 4^d^	Follow‐up	Information
	33. Discussion of the organization of KDA 4	Actors in KDAs	24 min	Knowledge dissemination Patient Engagement Project	Discussion of KDA 4 programme and of each member's role, and instructions to attend it	Implication
	34. Discussion of impacts of PE and validation of analysis and evaluation of the Project	Participants in evaluation of Patient Engagement Project	1 h 53 min	Validation of results and interpretations Patient Engagement Project	Spontaneous discussion on experiences of patient engagement	Implication
	Other					
Meeting # 12 6 September 2017/2 h 51 m /8 members/Research Institute of the MUHC	35. Discussion on KDA 4 and information on development and publications	Actors in KDAs	14 min	Knowledge dissemination Patient Engagement Project	Follow‐up on KDA 4; Summary of recent publications and presentations at conferences	Information
	36. Overview of Team's functions in Mentorship Chair	Stakeholders in decision making	37 min	Functioning Subproject 4^d^	Common understanding of Team's functions in Mentorship Chair	Implication
	37. Validation of analysis and evaluation of the Project and design of evaluation of meetings in next phases	Participants in evaluation of Patient Engagement Project	1 h 29 min	Validation of data collection instrument Patient Engagement Project	Spontaneous discussion on experiences of patient engagement	Collaboration
	Other		38 min			
KDA 1						
Date: 30 November 2016				Duration: 1 h		
Attendance: 20 health‐care providers and academics				Venue: Conference room of the MUHC		
Content: DL, BL, and a Team member presented on the functioning of the Patient Engagement Project and the results of the needs assessment						
KDA 2						
Date: 1 December 2016				Duration: 3 h		
Attendance: 5 representatives of the sponsoring pharmaceutical company				Venue: Conference room of the MUHC		
Content: DL, BL, KE, and a Team member presented on the functioning of the Patient Engagement Project and the results of the needs assessment						
KDA 3						
Date: 1 December 2016				Duration: 3 h		
Attendance: 70 community members (including 6 Team members)				Venue: bar in the Montreal Gay Village		
Content: DL, BL, and a Team member presented on the role of adherence in preventing disease progression and forward sexual transmission of HIV, and two other Team members provided written testimonials on their experience of treatment adherence and therapeutic success.						
KDA 4						
Date: 11 May 2016				Duration: 8 h		
Attendance: 20 people (academics, community organizations actors, health‐care providers, and 3 Team members)				Venue: Conference room on university campus		
Content: During a national conference for the advancement of francophone science, KE, IT, DL and two Team members presented on patient engagement in the I‐Score Study, discussed the respective role of clinicians’ and patients’ perspectives in HIV clinical research and reflected on their role in the research process. They discussed these questions with other invited speakers (academics, community organizations representatives and health‐care providers).						

KDA, knowledge dissemination activity; MUHC, McGill University Health Centre; PLHIV, people living with HIV; PRO, patient‐reported outcome measure.

a Subproject 1: Needs assessment concerning preferences and needs for the I‐Score PRO consisting of focus groups with HIV clinicians.

b Subproject 2: Effectiveness/Implementation Hybrid Study evaluating the application of the I‐Score PRO measure in clinical practice.

c Subproject 3: Project for the development of recommendations by and for HIV patients and clinicians to manage ART adherence barriers.

d Subproject 4: Application by principal investigator for a Canadian Institutes of Health Research Strategy for Patient‐Oriented Research Mentorship Chair in Patient‐Oriented Research and Innovative Clinical Trials (November 2016).

## 3 RESULTS

### 3.1 Nature of PE

The nature of PE includes details on the meetings’ discussion themes, functions held by members and the KDAs. Table 3 shows meeting, discussion and KDA characteristics summarized from observational notes.

A total of twelve meetings took place during the evaluation period. Meetings 1‐2 were devoted to a qualitative needs assessment for the I‐Score PROM during which sex‐specific focus groups were facilitated by IT and observed by DL. Focus groups lasted about two hours and took place in a room provided by a partner community organization.

Meetings 3‐12 were regular meetings facilitated by DL, except for two discussions (29 and 31) which were co‐facilitated by Team members. Meetings lasted an average of 2 h 39 min. Meeting 3 took place at the MUHC (hospital), subsequent meetings, in a community centre, and Meeting 12, at the Research Institute of the MUHC. For these meetings, as mentioned, DL prepared agendas which were sent to members electronically two days in advance. In Meetings 3 to 6, there were an average of 4.75 distinct discussions (ie, discussion themes) per meeting lasting an average of 29 minutes each. In Meetings 7 to 10, discussions were less numerous and lasted longer (average of 2.75 discussion themes per meeting lasting an average of 50 minutes each). Meetings 11‐12 had 3 discussions each, lasting an average of 46 minutes. Meetings were attended by 6 (Meeting 6) to 9 (Meetings 3, 4, 5 and 10) members; three members (03, 04 and 06) attended all meetings, and other members missed one (02, 12, 15) or three (01, 08, 16) meetings; one member (07; a woman residing about an hour from Montreal) left the Project after Meeting 3.

During these meetings, 37 discussions on different themes were held with the Team and determined its functions. First, members’ discussions engaged them in *decision‐making processes,* in two‐thirds of meetings (Meetings 3, 4, 5, 6, 7, 9, 11 and 12). Relevant discussions during these meetings mainly concerned the I‐Score Study (six discussions) and the functioning of PE (seven discussions). These discussions not only informed members but asked their opinion about research processes and concepts associated with the I‐Score Study. Their input often served to improve/validate data collection instruments and research results, and, more specifically, make decisions concerning the development of the I‐Score PROM and the organization of PE. Additionally, members were actively involved in the expansion of the I‐Score Study into a broader research programme: they gained an advisory status on four subprojects of the I‐Score Study and eight Team discussions were held on these subprojects (Meetings 5, 6, 7, 9, 10 and 11). Subprojects 1 and 4 were introduced to members by investigators, while Subprojects 2 and 3 emerged from members’ suggestions (see Table 3). Also, one Team member with a strong academic background became a co‐investigator/partner on Subprojects 2, 3 and 4.

Second, members collaborated in a total of four *knowledge dissemination activities*. They discussed their organization or other related processes (eg, developing academic publications) in over 40% of meetings (Meetings 7, 9, 10, 11 and 12). The first KDA took place about a year after the Project began. KDAs lasted between one and eight hours (average = 3.75 hours) and took place in different settings. During KDAs, investigators and Team members presented their respective perspectives on ART adherence (KDAs 1, 2, 3 and 4) and on PE and research (KDA 4). These presentations were adapted to their respective audience. Between 5 (KDA 2) and 70 (KDA 4), people were present, including health‐care professionals (KDAs 1 and 4), academics (KDAs 1 and 4), sponsors (KDA 2) and community members and actors (KDAs 3 and 4). Two members attended and one member presented at KDA 1;^68^ one member presented at KDA 2; six members attended and one member presented at KDA 3;^69^ and three members attended and two members presented at KDA 4.^70^

Third, members *participated in research* at all meetings by taking part in: focus groups aimed at generating data for the I‐Score PROM needs assessment (Meetings 1 and 2); or focus groups to validate analyses generated by the I‐Score Study, the PE Project or Subproject 1 (Meetings 4, 5, 6, 8 and 9). Also, members participated in the evaluation, which involved individually filling out satisfaction surveys (Meetings 3, 5, 7, 9 and 11) and having Team discussions guided by the survey's open‐ended questions (Meetings 4, 6, 8, 10). Finally, they discussed the evaluation results for validation purposes and to involve them in the evaluation process (Meetings 11 and 12).

Results for five studies which received Team feedback have been published: the needs assessment conducted during Meetings 1 and 2,^71^ a review of HIV‐specific PROMs,^42^ a qualitative synthesis to produce the PROM's conceptual framework^43^ and studies on clinicians’ perceptions of patients’ needs regarding the I‐Score PROM (Subproject 1).^72^, ^73^ Qualitative research with the Team defined and confirmed PLHIV's needs for the I‐Score PROM and the Study subprojects and contextualized I‐Score results within PLHIV's lived experience. As to the PE Project evaluation, members demanded amendments to the satisfaction survey (Discussion 37). They wished the latter would better reflect the impacts they perceived of PE in many dimensions of their lives. For example, they wished for the addition of scales to rate their relationship with providers, the quality of their physical, sexual, emotional, relational and professional lives, and their comfort with HIV disclosure. The amended survey will be used in the next phase of the Project.

### 3.2 Levels of PE achieved

Meetings 1 and 2 (focus groups) were consultations. In Meetings 3 to 6, levels of engagement oscillated between information (26%; 5/19 discussions), consultation (42%; 8/19) and implication (32%; 6/19). These levels increased over time: Meetings 7 to 10 included information (30%; 3/10), consultation (10%; 1/10), implication (40%; 4/10) and collaboration (20%; 2/10). Meetings 11 and 12 reached the levels of information (33%; 2/6), implication (50%; 3/6) and collaboration (17%; 1/6).

### 3.3 Impacts of PE

This section presents the descriptive statistics of the satisfaction survey items and the results of the thematic analysis, offering a patient perspective on PE.

#### 3.3.1 Satisfaction

Table 2 presents descriptive statistics on members’ quantitative responses to the satisfaction survey. In Meeting 3, when the survey was first administered, average satisfaction ratings for “learning new information” (*M *=* *3.2), “learning new skills” (*M *=* *3.8) and the “venue/facility” (*M *=* *3.8) were lowest. After Meeting 3, members expressed high and stable rates of satisfaction on all aspects considered (range: 3‐5; *M *=* *4.5; SD = 0.73), especially with regard to “interest,” “relevance,” “enjoyment” and the “facilitation.”

#### 3.3.2 Member‐identified impacts

The thematic analysis of the qualitative survey items and transcripts generated four member‐identified impacts of PE, described below, which help contextualize members’ satisfaction ratings. Table 4 presents these themes and illustrative member quotes.

**Table 4:** Meeting and survey comments exemplifying member‐identified impacts of patient engagement in the Project

Member‐identified impacts	Example comments from the satisfaction surveys^a^	Example comments from the transcriptions
Positive interactions	*Things that went well* ”Exchanging with others” ”The flow of communication” ”Good interpersonal contact” ”Flow of conversation, people voicing opinions” ”Turn to speak” ”Flow of conversation, humor” *Things members will remember* ”All participants having their say” *Things members learned about themselves* ”I have to keep on improving the way I receive others’ opinions”	03: The fact of coming to Team meetings is as if we found a human aspect that is lost, say, at the clinic. HIV often equals sickness, treatment, it's a cold field. But here, we are on the human, warm side again. 04: Personally, I am feeling valued. Before, if I participated in research, I felt like a guinea pig, I felt used. Now, I am proud that I am dealing with experts, that they consider our perspective. I feel that I have something to bring. Us, we bring the experience, researchers, they bring their expertise. 06: Meeting here enriched the way I intervene with [other PLHIV]. I learned other ways of doing things, of understanding. Before, I would say things like this, just throw them around. Now, I have a conviction and arguments, I see clearer.
Co‐learning	*Things members will remember* ”Elements or factors that can impede regular pill‐taking” ”The new definition for adherence” ”Iatrogenic effects of medication” ”‘Domino effects.’ They are changes that happen from time to time in my life and that have consequences for my adherence to ART, given the uncertainty of the future” *Things members learned about themselves* ”I have a simple experience but it can still help others” ”[Good] pressure to put good health practices in place (sport, exercise, supplements)” *Things that may impact their practices* ”To better discuss with my clinician and to get informed without shame”	03: I am realizing I'm crossing a moment of weathering (concept discussed in former meetings) right now, because I think that I'm tired of struggling to take ART. *[spontaneous usage of a concept discussed in a past meeting]* 15: We learned a lot of things from the meeting material and from other members. For example, we learned about clinicians’ approaches. I thought I had bad luck or that they did not listen to me carefully. When the others spoke about it, it changed, because we noticed and named problems that exist everywhere in clinicians’ approaches. 12: I didn't know if there was an interaction between my ART and calcium. But [06] brought the topic up, about iron, and I asked my clinician. He realized there could be an interaction, and said I have to take [ART] at least two hours after taking the calcium. 15: In the beginning, researchers gave importance to our gender and age. It seemed to be a priority. There are men and women, it is a good thing. And among us members, not everybody has the same problems. By sharing together, we learned from our respective stories and problems, so this diversity should not be neglected.
Self‐determination	*Things that went well* ”We chose the name for the group” *Things that could be improved* ”The place of the meeting” ”We should begin at 5 PM” ”It would be good to have individual lunch boxes” ”To improve the presentation of documents, as some charts are unreadable” *Things members will remember* ”The role I play in the Project” ”The importance of the committee”	08: Meeting here, close to the place where I met people from [a specific organization] and formed support, buddy systems, makes a difference. I never had to hide here, we can be ourselves, speak openly. […] And this room is big and warm and beautiful. Here our thoughts can flow and feel free. 06: We do not want to control or force ideas on researchers, because we do not have this knowledge. We concentrate on what they expect from us: they consult us about our ideas and our experience, and then they see how this fits into their way of doing things. 06: When we looked for our name, how to qualify us, I thought that this exercise clarified many things in what we were doing. We named our expectations, our objectives, and what is engagement for us. It helps us understand. 04: When we wrote the support letter, we thought it was not in our image at first, it did not reflect us. We worked together and we came to an agreement on the language to use. 06: Engagement applies to daily life as well. We were informed, and we do things outside of research when the information may apply. Engagement is part of a broader set of activities for patients. We define from our own situation what is engagement for us.
Collective management of confidentiality	*Things members learned about themselves* ”I feel comfortable talking about certain aspect of my life as an HIV‐positive person” (Meeting # 1) ”I have no problem accepting or disclosing my HIV status” (Meeting # 5) ”Sometimes I do not open up to interveners because I'm afraid to be discriminated” (Meeting # 5) *Things that may impact their practices* ”I am questioning to which extent I want to disclose publicly or not my status (during workshops, for example)” (Meeting # 1)	06: Some [other PLHIV] say: “Oh, she testified, she spoke,” and they feel entitled to ask me to disclose [my HIV status] everywhere. […] They forget that at some point, I cannot always do it, and it is not my responsibility to talk for you. 15: Some members among us have played a very active role and presented publicly. It relaxed the atmosphere, provided models, and led the others to get on board. 03: I had been recently infected when the Project began, and nobody knew about my status. I thought: “Oh my God! I will have to talk with the others!” Now, I feel good, the meetings helped me. If you had asked me to present [at KTA 4] at the beginning, I would have said no. Now it's alright, I'm happy to do it.

ART, antiretroviral therapy; KDA, knowledge dissemination activity.

a A majority of members’ survey comments (117/124, 94%) were coded under these impacts.

*Positive interactions:* This impact refers to mention of positive and rewarding elements of interactions between members, with investigators, or with other PLHIV, in terms of respect, conviviality and mutual support. Members indicated feeling listened to and valued by investigators, or when sharing knowledge with other Team members or PLHIV. This was said to partly maintain their motivation to participate in the Project. This theme is consistent with their high and stable satisfaction ratings, notably on the “interest,” “enjoyment” and “meeting with people” items.

*Co‐learning* captures mention of collective learning around health. In the open‐ended satisfaction survey questions, members described learning relevant care‐ or research‐related concepts and claimed to subsequently use them. As mentioned above, ratings on the two quantitative survey items concerned with learning were among the lowest at Meeting 3 but, afterwards, they reached high levels.

According to members, they mostly learned by exchanging together on HIV‐related social experiences (eg, stigma at work, family support), medical aspects (eg, treatments, medical facts, engagement in health care) and current events (HIV‐related or not). Approximately half of all Team discussions (51%; 19/37) were spontaneous, non‐research‐centred and devoted to members’ experiences and concerns.

Among their learnings, members most frequently mentioned improved communication skills: they learned to participate in discussions, listen to other members’ opinions and better communicate with care providers. They mentioned acquiring skills to situate the perspectives from which they spoke. As the Project evolved, these perspectives expanded beyond those of HIV patient or infection group to include those of parent, professional or patient of a non‐HIV‐specific provider (eg, physiotherapist), among others. This sensitivity to positioning stimulated discussions by raising questions about the experiences of others facing different circumstances (eg, a woman pondering how gay men experience HIV‐related stigma).

Most members also mentioned improving their ability to support other PLHIV (eg, friends, family members) with ART‐taking. Some mentioned increased health‐related or ART‐taking skills (eg, learning to recognize adherence barriers, such as side‐effects, and when to discuss these with their providers) and exchanged information on local clinics. A few members claimed to have improved their access to adapted care.

*Self‐determination* refers to members’ input concerning the organization of PE and their functions. Members made numerous suggestions in the satisfaction survey to improve accessibility and comfort during meetings. Importantly, average satisfaction for the “venue” survey item was relatively low for Meeting 3 (*M *=* *3.8), which took place in a hospital. It subsequently increased when meetings were immediately moved to a more convivial and accessible community‐based venue.

Members described their functions in Discussions 9, 13, 27 and 36, in a way that is consistent with the PE level of “collaboration” with investigators. For them, their role was to share their perspective on research‐related topics in a way and at a time that is efficient for investigators. They also decided that some members would assume specific and periodic responsibilities, as circumstances dictated (eg, facilitating key discussions or organizing KDAs).

The *collective management of confidentiality* refers to the collaborative management of concerns about confidentiality. The nature of the Project involved meetings with other PLHIV or presenting before various audiences (ie, KDAs). Members expressed different levels of comfort with disclosing personal information, including their HIV status. These tensions were discussed explicitly during Discussion 4, and when facing situations that could expose members, including academic publications (Discussions 7, 19 and 28), KDAs (Discussions 23, 30 and 33) and the preparation of a Team letter of support for Subproject 4 (Discussion 27).

Members decided that KDAs were important to transmit lessons learned and ensure the visibility and continuity of the Team, while fighting stigma. However, they agreed that member participation had to be completely voluntary and that KDAs had to be organized for audiences believed to be less likely to stigmatize PLHIV (eg, other PLHIV, health professionals and academics). Members more comfortable with disclosure participated in higher profile activities but expressed concern about “speaking for others.”

## 4 DISCUSSION

This paper presents a rare detailed description of a PE Project with PLHIV during its first phase (22 months) and results of its evaluation. This Project combined PE across the research cycle^31^, ^32^, ^33^ of a PROM development study, mainly through committee meetings and knowledge dissemination, with patient participation in complementary research. To avoid confusion, these components were clearly defined and explained to engaged PLHIV at consent. Our PE Project's evaluation addressed the documented lack of reporting on many aspects of PE,^10^, ^31^, ^32^, ^36^ offering a description of its nature, levels and impacts. It combined qualitative and quantitative data collection methods and involved patients it the evaluation, contributing to the limited research on engaged patients’ perspectives on PE^25^, ^29^ and its impacts.^35^ The evaluation's results contributed to knowledge of what attracts patients to PE, maintains their interest and increases their ability to engage in research.^32^, ^33^ Overall, they deepened our understanding of PLHIV's needs regarding both the PE Project and the I‐Score Study.

As in other PE initiatives,^55^ we observed that PE levels raised over time. Meetings 1 to 6 were characterized by an emphasis on “information” and/or “consultation,” while PE levels subsequently increased to include greater “collaboration” and deeper and lengthier discussions. Explanations include changes in the investigators’ requests of the Team, as the Study advanced; skills and knowledge gained through PE (eg, “co‐learning”); and greater interaction with investigators (eg, during the organization of KDAs). Furthermore, with time, one Team member became a co‐investigator on several I‐Score subprojects. This level of recognition of experiential knowledge and more active and influential role in research decision making can be empowering to patients.^74^ The range of PE also expanded over time, as members became involved in KDAs and I‐Score subprojects. For instance, the Team was involved in Subproject 4 (a successful application, in November 2016, for a Canadian Institutes of Health Research Strategy for Patient‐Oriented Research (CIHR/SPOR) Mentorship Chair), an activity that seldom benefits from patient input.^10^, ^30^, ^31^ PE may continue to expand in the future, given that the Mentorship Chair awarded to BL in February 2017 provides cohesion and continuity for the Team, allowing it to continue its work on the I‐Score Study and Chair‐affiliated projects.

Our results, nevertheless, indicate that within a same PE Project, patients can be engaged differently. Not all patients are able to invest the same time and energy in PE.^54^, ^75^ Members commented on the meeting attendance results by underlining how PE sometimes conflicted with unexpected events, their professional or personal responsibilities or other commitments. Engagement was also affected by HIV disclosure concerns, a documented challenge of PE among PLHIV.^24^, ^28^ Our findings on the “collective management of confidentiality” highlight the need for ongoing management of confidentiality issues. Such management meant that certain members limited their participation in meetings, KDAs and data collection activities, due to HIV disclosure concerns, and in some instances, members acted collectively. For example, they co‐authored a publication under their Team label.^71^ Involving members in KDAs, especially, required flexibility and trust, and, while some members actively participated (eg, presenting), others did not. Overall, the Team consistently sought to find workable solutions for members, in contexts of wanted or unwanted disclosure.

Our results suggest that members appropriated, to a certain extent, their roles and PE processes, so the Project could better fulfil their expectations. About half of discussions were not entirely research‐focussed: members appropriated discussions, focussing on (inter)personal outcomes (eg, improved communication skills, increased ability to support other PLHIV, enhanced health‐related skills). These patient‐appropriated discussions can be considered “pluralist” interactions, that is, interactions that do not reduce actors to a single dimension. Pluralist interactions are theorized as ways to foster more equality in PE and empower patients. They help uncover patients’ motivations with PE,^12^, ^37^, ^39^, ^55^, ^76^ concerns (eg, comorbidities, financial issues, etc.), values and understandings of health, illness and care distinct from investigators’, and to value their experiential knowledge.^77^, ^78^ Like other PE initiatives that seek rigorous reporting of methods,^45^, ^79^ we initiated the Project by applying qualitative research sampling methods, selecting patients from targeted populations to provide different perspectives (ie, HIV patients, members of HIV infection groups). However, pluralist interactions and our results on “co‐learning” illustrate how members did not always speak from the positions initially assigned by investigators. Rather, they reminded us that patients, like all individuals, hold multiple positions in society.^75^ More tools are needed to evaluate PE from engaged patients’ perspective and better account for the complexity of their needs.

Overall, these points echo the work of authors who argue for adapting PE to context and patients’ expectations and resources.^35^, ^75^, ^80^ Taken together, the four member‐identified impacts that we found highlight the importance of positive interactions and collaboration^81^ between patients, and paying attention to patients’ experiences.^35^ These principles could contribute to developing valuable PE approaches that address identified challenges of engaging PLHIV.^33^, ^36^, ^37^

### 4.1 Some limitations

The already conceptualized I‐Score Study provided the PE Project with initial funding, but there was no PE in its early design. This issue is documented in many PE projects that face insufficient funding or infrastructure to engage patients at early stages, when establishing research priorities and designing projects.^82^ Nevertheless, given the I‐Score Study's multiyear duration, it offered a valuable opportunity to initiate and improve PE. Furthermore, the infrastructure provided by the CIHR/SPOR Mentorship Chair has contributed to the sustainability of PE in our work.

Most Team members had some academic or clinical research experience, at least as participants, or had frequented at least one community organization. They generally appreciated the diversity in gender and ethnic backgrounds within the Team but underlined the absence of people who identify as trans, a group disproportionately affected by HIV and current injection drug users. Our recruitment and PE methods excluded PLHIV facing difficult circumstances. For example, during recruitment, some candidates withdrew because they felt too burdened with mental health issues. More could be done to reach and include marginalized individuals, especially recently diagnosed, young or disengaged PLHIV,^83^ given that studies show that marginalized PLHIV are often willing to participate when asked.^84^, ^85^

Alternative research and PE methods (eg, online KDAs, individual interviews, collaboration with community‐based organizations) could be explored, as continuous PE in a group setting might not be suitable to all PLHIV. Yet, most members mentioned, during meetings, having experienced moments of significant vulnerability before or during the Project, in the form of comorbidities, disabilities, sexual or physical violence, and socio‐economic inequality. Some mentioned having overcome, learned from and wanting to share lessons learned from these hardships by participating in the Project. PE projects often depend on patients’ will and abilities to share knowledge gained from positive and/or negative experiences (REF). Yet, it may take time for patients to do so.

Limitations of the evaluation also include the fact that members may have biased their input and comments by favouring social desirability and groupthink,^49^ vis‐à‐vis investigators and other members, over addressing contentious issues. As in other studies of PE with PLHIV,^86^, ^87^ one person (DL) was mainly responsible for facilitating and maintaining PE, taking meeting notes and conducting the analyses, which could have biased our results. Nevertheless, analyses and results were regularly discussed with other investigators and with Team members for validation, including discussions on the perceived impacts of PE.

## 5 CONCLUSION

This mixed method evaluation offers a detailed account of a project to engage PLHIV in a PROM development study, contributing to the limited research on patients’ perspectives on PE. Overall, the low attrition rate and the high satisfaction scores suggest that the Project was a positive experience for members.

The beginning of the Project was characterized by adjustments and information‐ or consultation‐oriented discussions with engaged PLHIV. With time, the level of PE increased: discussions focused more on collaboration; KDA's involving Team members were organized; and the Team's contribution to research expanded with the development of subprojects and partnerships. The patients stressed impacts of PE in terms of positive interactions and collaboration between members.

Our results highlight the importance of more patient‐centric practices and comprehensive evaluations of PE. This would improve understanding of the processes and outcomes of PE to also recognize patient‐valued ramifications beyond research.

## References

[1] Canadian Institutes of Health Research . Patient Engagement. 2014; http://www.cihr-irsc.gc.ca/e/45851.html. Accessed March 31, 2017.

[2] Snyder H , Engström J . The antecedents, forms and consequences of patient involvement: a narrative review of the literature. Int J Nurs Stud. 2016;53:351‐378. 26602069 10.1016/j.ijnurstu.2015.09.008

[3] Bruni RA , Laupacis A , Levinson W , Martin DK . Public involvement in the priority setting activities of a wait time management initiative: a qualitative case study. Health Serv Res. 2007;7:186. 10.1186/1472-6963-7-186 PMC2238747 18021393

[4] Diaz Del Campo P , Gracia J , Blasco JA , Andradas E . A strategy for patient involvement in clinical practice guidelines: methodological approaches. Qual Saf. 2011;20(9):779‐784. 10.1136/bmjqs.2010.049031 21460393

[5] Grande SW , Faber MJ , Durand MA , Thompson R , Elwyn G . A classification model of patient engagement methods and assessment of their feasibility in real‐world settings. Patient Educ Couns. 2014;95(2):281‐287. 24582473 10.1016/j.pec.2014.01.016

[6] Forbat L , Hubbard G , Kearney N . Patient and public involvement: models and muddles. J Clin Nurs. 2009;18(18):2547‐2554. 19207798 10.1111/j.1365-2702.2008.02519.x

[7] Pomey MP , Flora L , Karazivan P , et al. Le “Montreal model”: enjeux du partenariat relationnel entre patients et professionnels de la santé. Sante Publique. 2015;27(1):41‐50. 26168616

[8] Shippee ND , Domecq Garces JP , Prutsky Lopez GJ , et al. Patient and service user engagement in research: a systematic review and synthesized framework. Health Expect. 2015;18(5):1151‐1166. 23731468 10.1111/hex.12090 PMC5060820

[9] Carman KL , Dardess P , Maurer M , et al. Patient and family engagement: a framework for understanding the elements and developing interventions and policies. Health Aff. 2013;32(2):223‐231. 10.1377/hlthaff.2012.1133 23381514

[10] Domecq JP , Prutsky G , Elraiyah T , et al. Patient engagement in research: a systematic review. Health Serv Res. 2014;14(1):1‐9. 10.1186/1472-6963-14-89 PMC3938901 24568690

[11] Concannon TW , Meissner P , Grunbaum JA , et al. A new taxonomy for stakeholder engagement in patient‐centered outcomes research. J Gen Intern Med. 2012;27(8):985‐991. 22528615 10.1007/s11606-012-2037-1 PMC3403141

[12] Mallery C , Ganachari D , Fernandez J , Smeeding L , Robinson S , Moon M . Innovative Methods in Stakeholder Engagement: An Environmental Scan. Rockville, MD: Agency for Healthcare Research and Quality; 2012.

[13] National Health Research Institute . Briefing Notes for Researchers: Involving the Public in NHS, Public Health and Social Care Research. Eastleigh: National Health Research Institute; 2012.

[14] Mosconi P , Colombo C , Satolli R , Liberati A . PartecipaSalute, an Italian project to involve lay people, patients’ associations and scientific‐medical representatives in the health debate. Health Expect. 2007;10(2):194‐204. 17524012 10.1111/j.1369-7625.2007.00444.x PMC5060390

[15] Oliver SR , Rees RW , Clarke‐Jones L , et al. A multidimensional conceptual framework for analysing public involvement in health services research. Health Expect. 2008;11(1):72‐84. 18275404 10.1111/j.1369-7625.2007.00476.x PMC5060424

[16] Alberta Health Services . A resource toolkit for engaging patient and families at the planning table. 2012; http://www.albertahealthservices.ca/assets/info/pf/pe/if-pf-pe-engage-toolkit.pdf. Accessed 22 November, 2017.

[17] Kirwan JR , de Wit M , Frank L , et al. Emerging guidelines for patient engagement in research. Value Health. 2017;20(3):481‐486. 28292494 10.1016/j.jval.2016.10.003

[18] Bellows M , Kovacs Burns K , Jackson K , Surgeoner B , Gallivan J . Meaningful and effective patient engagement: what matters most to stakeholders. Patient Exp J. 2015;2(1):18‐28.

[19] Nelimarkka M , Nonnecke B , Krishnan S , et al. Comparing Three Online Civic Engagement Platforms using the “Spectrum of Public Participation” Framework. Paper presented at: The Internet, Policy & Politics Conferences, 2014; Oxford.

[20] Brett J , Staniszewska S , Mockford C , et al. Mapping the impact of patient and public involvement on health and social care research: a systematic review. Health Expect. 2014;17(5):637‐650. 22809132 10.1111/j.1369-7625.2012.00795.x PMC5060910

[21] Oliver S , Clarke‐Jones L , Rees R , et al. Involving consumers in research and development agenda setting for the NHS: developing an evidence‐based approach. Health Technol Assess. 2004;8(15):1‐148. 10.3310/hta8150 15080866

[22] Workman T , Maurer M , Carman K . Unresolved tensions in consumer engagement in CER: a US research perspective. J Comp Eff Res. 2013;2(2):127‐134. 24236555 10.2217/cer.13.6

[23] Barello S , Graffigna G , Vergni E , Bosio AC . The challenges of conceptualizing patient engagement in health care: a lexicographic literature review. J Particip Med. 2014;6:e9.

[24] UNAIDS . The Greater Involvement of People Living with HIV (GIPA). 2007; http://data.unaids.org/pub/briefingnote/2007/jc1299_policy_brief_gipa.pdf. Accessed March 26, 2018.

[25] Morolake O , Stephens D , Welbourn A . Greater involvement of people living with HIV in health care. J Int AIDS Soc. 2009;12(4):1‐7. 10.1186/1758-2652-12-4 PMC2664321 19284672

[26] Mugavero MJ , Norton WE , Saag MS . Health care system and policy factors influencing engagement in HIV medical care: piecing together the fragments of a fractured health care delivery system. Clin Infect Dis. 2011;52(Suppl 2):S238‐S246. 21342913 10.1093/cid/ciq048 PMC3106258

[27] Flickinger TE , Saha S , Moore RD , Beach MC . Higher quality communication and relationships are associated with improved patient engagement in HIV care. J Acquir Immune Defic Syndr. 2013;63(3):362‐366. 23591637 10.1097/QAI.0b013e318295b86a PMC3752691

[28] Varni SE , Miller CT , McCuin T , Solomon S . Disengagement and engagement coping with HIV/AIDS stigma and psychological well‐being of people with HIV/AIDS. J Soc Clin Psychol. 2012;31(2):123‐150. 22611302 10.1521/jscp.2012.31.2.123 PMC3355931

[29] Rhodes SD , Malow RM , Jolly C . Community‐based participatory research: a new and not‐so‐new approach to HIV/AIDS prevention, care, and treatment. AIDS Educ Prev. 2010;22(3):173‐183. 20528127 10.1521/aeap.2010.22.3.173 PMC3282157

[30] Brizay U , Golob L , Goberman D , Gogolishvili D , Bird M . Community‐academic partnerships in HIV‐related research: a systematic literature review of theory and practice. J Int AIDS Soc. 2015;18(1):1‐12. 10.7448/IAS.18.1.19354 PMC4309828 25630823

[31] Concannon TW , Fuster M , Saunders T , et al. A systematic review of stakeholder engagement in comparative effectiveness and patient‐centered outcomes research. J Gen Intern Med. 2014;29(12):1692‐1701. 24893581 10.1007/s11606-014-2878-x PMC4242886

[32] Esmail L , Moore E , Rein A . Evaluating patient and stakeholder engagement in research: moving from theory to practice. J Comp Eff Res. 2015;4(2):133‐145. 25825842 10.2217/cer.14.79

[33] Manafo E , Petermann L , Mason‐Lai P , Vandall‐Walker V . Patient engagement in Canada: a scoping review of the ‘how’ and ‘what’ of patient engagement in health research. Health Res Policy Syst. 2018;16(1):5. 29415734 10.1186/s12961-018-0282-4 PMC5804082

[34] Staniszewska S , Brett J , Mockford C , Barber R . The GRIPP checklist: strengthening the quality of patient and public involvement reporting in research. Int J Technol Assess Health Care. 2011;27(4):391‐399. 22004782 10.1017/S0266462311000481

[35] Crocker JC , Boylan AM , Bostock J , Locock L . Is it worth it? Patient and public views on the impact of their involvement in health research and its assessment: a UK‐based qualitative interview study. Health Expect. 2017;20(3):519‐528. 27338242 10.1111/hex.12479 PMC5433537

[36] Brett J , Staniszewska S , Mockford C , et al. A systematic review of the impact of patient and public involvement on service users, researchers and communities. Patient. 2014;7(4):387‐395. 25034612 10.1007/s40271-014-0065-0

[37] Bradley M , Braverman J , Harrington M , Wicks P . Patients’ motivations and interest in research: characteristics of volunteers for patient‐led projects on PatientsLikeMe. Res Involv Engagem. 2016;2(1):33. 29507767 10.1186/s40900-016-0047-6 PMC5831886

[38] Jennifer H , Katherine C , Stephen L , Kerryn P . Appealing to altruism is not enough: motivators for participating in health services research. J Empir Res Hum Res Ethics. 2012;7(3):84‐90. 22850146 10.1525/jer.2012.7.3.84

[39] Hearld KR , Hearld LR , Hall AG . Engaging patients as partners in research: Factors associated with awareness, interest, and engagement as research partners. SAGE Open Med. 2017;5(10):205031211668670. 10.1177/2050312116686709 PMC5308537 28228949

[40] Forbat L , Cayless S , Knighting K , Cornwell J , Kearney N . Engaging patients in health care: an empirical study of the role of engagement on attitudes and action. Patient Educ Couns. 2009;74(1):84‐90. 18790594 10.1016/j.pec.2008.07.055

[41] Engler K , Lessard D , Toupin I , Lènàrt A , Lebouché B . Engaging stakeholders into an electronic patient‐reported outcome development study: on making an HIV‐specific e‐PRO patient‐centered. Health Policy Technol. 2017;6(1):59‐66.

[42] Engler K , Lessard D , Lebouche B . A review of HIV‐specific patient‐reported outcome measures. Patient. 2017;10(2):187‐202. 27637488 10.1007/s40271-016-0195-7

[43] Engler K , Lenart A , Lessard D , Toupin I , Lebouche B . Barriers to antiretroviral therapy adherence in developed countries: a qualitative synthesis to develop a conceptual framework for a new patient‐reported outcome measure. AIDS Care. 2018;30(sup1):17‐28. 29719990 10.1080/09540121.2018.1469725

[44] Bitton A , Onega T , Tosteson ANA , Haas JS . Toward a better understanding of patient‐reported outcomes in clinical practice. Am J Manag Care. 2014;20(4):281‐286. 24884859 PMC4083494

[45] Morgan H , Thomson G , Crossland N , Dykes F , Hoddinott P , Team Bs . Combining PPI with qualitative research to engage ‘harder‐to‐reach’ populations: service user groups as co‐applicants on a platform study for a trial. Res Involv Engagem. 2016;2:7. 29062508 10.1186/s40900-016-0023-1 PMC5611582

[46] Doria N , Condran B , Boulos L , Curtis MD , Dowling L , Levy A . Sharpening the focus: differentiating between focus groups for patient engagement vs. qualitative research. Res Involv Engagem. 2018;4:19. 29983994 10.1186/s40900-018-0102-6 PMC6016125

[47] Pandya‐Wood R , Barron DS , Elliott J . A framework for public involvement at the design stage of NHS health and social care research: time to develop ethically conscious standards. Res Involv Engagem. 2017;3:6. 29062531 10.1186/s40900-017-0058-y PMC5611655

[48] Evans R , Kotchetkova I . Qualitative research and deliberative methods: promise or peril? Qual Res. 2009;9(5):625‐643.

[49] Morgan H , Hoddinott P , Thomson G , et al. Benefits of Incentives for Breastfeeding and Smoking cessation in pregnancy (BIBS): a mixed‐methods study to inform trial design. Health Technol Assess. 2015;19(30):1‐522, vii‐viii. 10.3310/hta19300 PMC4780978 25897655

[50] Nachega JB , Uthman OA , Peltzer K , et al. Association between antiretroviral therapy adherence and employment status: systematic review and meta‐analysis. Bull World Health Organ. 2015;93(1):29‐41. 25558105 10.2471/BLT.14.138149 PMC4271680

[51] Marshall MN . Sampling for qualitative research. Fam Pract. 1996;13(6):522‐525. 9023528 10.1093/fampra/13.6.522

[52] Boivin A , Lehoux P , Burgers JS , Grol RP . What are the key ingredients for effective public involvement in health care improvement and policy decisions? A randomized trial process evaluation. Milbank Q. 2014;92:319‐350. 24890250 10.1111/1468-0009.12060 PMC4089374

[53] Institut National de Santé Publique du Québec . Rapport Intégré: Épidémiologie des Infections Transmissibles Sexuellement et par le Sang au Québec. Quebec: Gouvernement du Québec; 2012.

[54] Truman C , Raine P . Involving users in evaluation: the social relations of user participation in health research. Crit Public Health. 2001;11(3):215‐229.

[55] Rhodes P , Nocon A , Booth M , et al. A service users’ research advisory group from the perspectives of both service users and researchers. Health Soc Care Community. 2002;10(5):402‐409. 12390226 10.1046/j.1365-2524.2002.00376.x

[56] Creswell JW . Research Design: Qualitative, Quantitative, and Mixed Methods Approaches. Thousand Oaks: SAGE Publications; 2014.

[57] LeCompte M , Schensul J . Designing and Conducting Ethnographic Research: an Introduction. 2010.

[58] Lassiter L . Collaborative ethnography and public anthropology. Curr Anthropol. 2005;46(1):83‐106.

[59] Lewis SJ , Russell AJ . Being embedded: a way forward for ethnographic research. Ethnography. 2011;12(3):398‐416.

[60] Pope C . Conducting ethnography in medical settings. Med Educ. 2005;39(12):1180‐1187. 16313576 10.1111/j.1365-2929.2005.02330.x

[61] Manchester Beacon . Engagement Evaluation Guide. Manchester: Manchester Beacon; 2016.

[62] Applebaum RA , Straker JK , Geron SM . Assessing Satisfaction in Health and Long Term Care: Practical Approaches to Hearing the Voices of Consumers. New York: Springer Publishing Company; 2000.

[63] Boyatzis RE . Transforming Qualitative Information: Thematic Analysis and Code Development. Thousand Oaks, CA: Sage Publications; 1998.

[64] Guest G , MacQueen KM , Namey EE . Applied Thematic Analysis. Los Angeles: Sage Publications; 2012.

[65] Van der Maren J . Méthodes de Recherche Pour l’éducation, 2e édn Paris: De Boeck & Larcier; 1996.

[66] Long T , Johnson M . Rigour, reliability and validity in qualitative research. Clin Effect Nurs. 2000;4(1):30‐37.

[67] Noble H , Smith J . Issues of validity and reliability in qualitative research. Evidence Based Nurs. 2015;18(2):34. 10.1136/eb-2015-102054 25653237

[68] Lessard D , Vicente S , Lebouché B . ART adherence from the patient perspective. Chronic and Viral Illness Service Academic Rounds; November 30, 2016, 2016; McGill University Health Centre, Montreal, Canada.

[69] Lessard D , Lebouché B , Toupin I , Risse D . Dialogue entre cliniciens, patients‐experts et le grand public sur les nouvelles réalités du traitement et de la prévention du VIH : Accès et barrières aux traitements antirétroviraux et à la PrEP. Infrastructure Transfert de Connaissances ‐ Réseau Sida/MI du FRSQ; December 1st, 2016, 2016.

[70] Lessard D , Lebouché B , Engler K , Toupin I , Vicente S . L'engagement des patients et des acteurs clés dans la recherche sur le VIH: Méthodes et retombées. Association francophone pour le savoir; May 11, 2017, 2017; Montréal, Canada.

[71] Lessard D , Engler K , Toupin I , Team I‐SC , Lebouché B . HIV patients’ perceptions of antiretroviral therapy adherence in relation to subjective time: imprinting, domino effects and future shadowing. J Int Assoc Provid AIDS Care. 2017;1:1‐8. 10.1177/2325958218759208 PMC6748544 29473484

[72] Toupin I , Engler K , Lessard D , et al. Developing a patient‐reported outcome measure for HIV care on perceived barriers to antiretroviral adherence: assessing the needs of HIV clinicians through qualitative analysis. Qual Life Res. 2018;27(2):379‐388. 29027607 10.1007/s11136-017-1711-5

[73] Toupin I , Engler K , Lessard D , et al. Patient profiles as organizing HIV clinicians’ ART adherence management: a qualitative analysis. AIDS Care. 2018;30(2):207‐210. 28764563 10.1080/09540121.2017.1360995

[74] Sacristan JA , Aguaron A , Avendano‐Sola C , et al. Patient involvement in clinical research: why, when, and how. Patient Prefer Adherence. 2016;10:631‐640. 27175063 10.2147/PPA.S104259 PMC4854260

[75] Tritter JQ . Revolution or evolution: the challenges of conceptualizing patient and public involvement in a consumerist world. Health Expect. 2009;12(3):275‐287. 19754691 10.1111/j.1369-7625.2009.00564.x PMC5060496

[76] Thompson J , Bissell P , Cooper CL , Armitage CJ , Barber R . Exploring the impact of patient and public involvement in a cancer research setting. Qual Health Res. 2014;24(1):46‐54. 24277776 10.1177/1049732313514482 PMC4509885

[77] Gibson A , Britten N , Lynch J . Theoretical directions for an emancipatory concept of patient and public involvement. Health. 2012;16(5):531‐547. 22535648 10.1177/1363459312438563

[78] Green G . Power to the people: to what extent has public involvement in applied health research achieved this? Res Involv Engagem. 2016;2:28. 29507763 10.1186/s40900-016-0042-y PMC5831888

[79] Maguire K , Britten N . “How can anybody be representative for those kind of people?” Forms of patient representation in health research, and why it is always contestable. Soc Sci Med. 2017;183:62‐69. 28463721 10.1016/j.socscimed.2017.04.049

[80] Tritter JQ , McCallum A . The snakes and ladders of user involvement: moving beyond Arnstein. Health Policy. 2006;76(2):156‐168. 16006004 10.1016/j.healthpol.2005.05.008

[81] Wendy H , Kate LD , Martin K . Value co‐creation through patient engagement in health care: a micro‐level approach and research agenda. Public Manage Rev. 2015;17(1):90‐107.

[82] Staniszewska S , Jones N , Newburn M , Marshall S . User involvement in the development of a research bid: barriers, enablers and impacts. Health Expect. 2007;10(2):173‐183. 17524010 10.1111/j.1369-7625.2007.00436.x PMC5060394

[83] Burchell AN , Gardner S , Light L , et al. Engagement in HIV care among persons enrolled in a clinical HIV cohort in Ontario, Canada, 2001‐2011. Implement Operat Res. 2015;70(1):e10‐e19. 10.1097/QAI.0000000000000690 PMC4623844 26322672

[84] Garber M , Hanusa BH , Switzer GE , Mellors J , Arnold RM . HIV‐infected African Americans are willing to participate in HIV treatment trials. J Gen Intern Med. 2007;22(1):17‐42. 17351837 10.1007/s11606-007-0121-8 PMC1824784

[85] Wendler D , Kington R , Madans J , et al. Are racial and ethnic minorities less willing to participate in health research? PLoS Med. 2006;3(2):e19. 16318411 10.1371/journal.pmed.0030019 PMC1298944

[86] Greco M , Carter M , Powell R , Sweeney K , Jollife J , Stead J . Impact of patient involvement in general practice. Educ Prim Care. 2006;17:486‐496.

[87] Carey MA , Smith MW . Enhancement of validity through qualitative approaches: incorporating the patient's perspective. Eval Health Prof. 1992;15(4):107‐114. 10117741 10.1177/016327879201500107

